# A proof‐of‐concept study on the effects of low total cfDNA content and solutions to increase the NIPT trisomy 21 detection rate

**DOI:** 10.1002/jcla.23035

**Published:** 2019-09-30

**Authors:** Yang Du, Ailing Chen, Rui Yang, Tao Zhou, Qin Zhou, Lan Yang, Juan Wang, Yan Hong, Chen Chen, Qian Wan, Lin Yang, Ying Chen

**Affiliations:** ^1^ Annoroad Gene Technology Co., Ltd Beijing China; ^2^ Central Lab Wuxi Maternity and Child Health Care Hospital Affiliated Wuxi Maternity and Child Health Care Hospital of Nanjing Medical University Wuxi, Jiangsu China; ^3^ Neonatal Department Wuxi Maternity and Child Health Care Hospital Affiliated Wuxi Maternity and Child Health Care Hospital of Nanjing Medical University Wuxi China

**Keywords:** low cfDNA content, non‐invasive prenatal testing, non‐reference‐based DV method, trisomy 21, *z*‐score

## Abstract

**Background:**

Non‐invasive prenatal testing (NIPT) is routinely used in clinical practice for fetal trisomy screening, but low total cfDNA content and low fetal fraction (LFF) are two factors that affect the detection rate. Samples with low total cfDNA or LFF usually end up with “no‐call” results, followed by the blood redraw and re‐testing, which is inconvenient for pregnant women and clinicians.

**Methods:**

We created mock trisomy 21 (T21) samples to investigate the effects of low total cfDNA with low LFF and possible solutions to increase their detection rate.

**Results:**

Samples with low total cfDNA resulted in the decreased unique reads number and increased duplication rate. Abnormal correlations between library concentration and raw reads number and the coverage fluctuation value, ZsdNorm, were also discovered, suggesting that low total cfDNA could lead to the overestimation of the library concentration. Additionally, a non‐reference‐based derivative value method (DV method) was evaluated and the data demonstrated that the detection sensitivity of trisomy 21 was increased from 33.33% (6/18) to 94.44% (17/18) in samples with 5% fetal fraction comparing with the *z*‐score approach, whereas for LFF (3.5%) group, the performance was raised from 0% to 35.29% (6/17).

**Conclusion:**

Low total cfDNA has significant impacts on NIPT performance by altering sequencing quality. The non‐reference‐based DV method could increase the T21 detection rate in samples with limited cfDNA content and 5% fetal fraction, but it was not as effective for those with LFF.

## 1 INTRODUCTION

Non‐invasive prenatal testing (NIPT) is an advanced screening method to detect fetal aneuploidy through cell‐free DNA (cfDNA) in maternal blood. The sensitivity and specificity of NIPT can reach nearly 100% for trisomy 21 (T21) and more than 90% for T18 and T13.^1^, ^2^ Therefore, it has been widely applied in clinical practice as an important prenatal screening method^3^, ^4^ to reduce the incidence of unnecessary invasive operation. However, about 2% of the pregnant women were found to have limited amount of circulating fetal DNA (low fetal fraction, LFF) in clinical practice.^5^ Previous reports indicated that LFF could lead to a much higher chance of discrepancy between NIPT and the invasive fetal karyotyping, and consequently, more false‐negative results would be obtained which in turn reduced the sensitivity of the test.^6^ Moreover, other factors such as low total cfDNA content, measured after the plasma DNA extraction, was also considered as one of the important reasons for NIPT test failure, because it could affect the library construction or reduce the DNA quantity in sequencing.^7^

In order to achieve a high screening accuracy, one of the most common strategies used in current clinical practice is to give uninformative NIPT results to women with LFF or low total cfDNA. This is referred as the test failure or more commonly known as “no‐call”. However, it was reported that pregnant women with “no‐call” results had an increased risk of aneuploidy.^8^ In addition, women with “no‐call” results were often advised to take a blood redraw and re‐testing, which brought inconvenience or even anxiety to women, and extra sequencing and labor costs to clinic laboratories. Furthermore, it was demonstrated that nearly thirty percent to fifty percent of the redraws would also receive “no‐call” results in the repeating test.^9^, ^10^ Therefore, for the benefit of both women and clinical practitioners, it would be helpful to provide more insight into such influencing factors and to find some new approaches to compensate the problem. However, most of the previous researches were focused on the LFF, the characteristic of low total cfDNA was ignored and its further influence on the results was still largely unknown. Herein, we created a set of mock T21 samples with low total cfDNA content and 3.5% or 5% fetal fraction (FF) to simulate the scenario and investigated its impacts on the NIPT results. Additionally, we carried out a proof‐of‐concept study by analyzing sequencing data with the non‐reference‐based derivative value method (DV method) and compared its results with the conventional *z*‐score approach to evaluate if the T21 detection rate could be increased in samples with low total cfDNA.

## 2 MATERIALS AND METHODS

### 2.1 Preparation of mock T21 Samples

In this study, two groups of mock T21 samples with low total cfDNA content plus low FF (3.5%) or normal FF (5%) were made from the peripheral blood of 18 T21 female patients and a healthy donor in the Affiliated Wuxi Maternity and Child Health Care Hospital of Nanjing Medical University. This study was approved by the Ethic Committee of the hospital, and informed written consents were obtained from all participants prior to blood sampling.

Briefly, 10 mL of peripheral blood from each T21 patients was drawn using the Streck cell‐free DNA BCT tube (Streck). After centrifugation at 1600 × *g* for 10 minutes at 4°C, the supernatant was collected and then subjected to a second centrifugation at 16 000 × *g* for 10 minutes, and the plasma (marked as plasma‐A) was collected and stored for further analysis. Meanwhile, 25 mL of peripheral blood was drawn from a healthy female and the plasma (marked as plasma‐B) was obtained with the aforementioned procedure. Then, each plasma‐A was mixed with the appropriate volume of plasma‐B to produce a series of mock T21 samples with 3.5% or 5% fetal fraction and the total volume of each mixture was 600 μL. Finally, 30% of each mock T21 sample was mixed (v/v) with 70% PBS buffer to mimic the situation of low total cfDNA. In total, we created 18 samples with 3.5% FF (named 3.5% T21 group), 18 samples with 5% FF (named 5% T21 group) and they all had low total cfDNA.

### 2.2 DNA Sequencing and data processing

After the DNA extraction using QIAseq cfDNA Extraction Kit (Qiagen), Qubit 3.0 system (Thermo Fisher Scientific) was used to test the cfDNA content of each sample. Library construction, quality control, and pooling were then sequentially carried out according to the user's manual. The concentration of each library was quantitated by the Qubit 3.0 and 12 libraries were pooled together and sequenced on the Ion proton platform as one batch. In total, three batches of pooled libraries were sequenced. Raw sequencing data were processed using an in‐house bioinformatics pipeline. Raw reads were sorted via barcodes, trimmed to keep the first 35 bp and filtered by the Phred score of Q ≥ 20. Next, reads with unique alignment (without mismatch) to human genome version 19 (hg19) were numbered and duplicated reads were removed and counted to calculate the overall duplication rate.

Chromosomal *z*‐score was calculated using the algorithm described in Qi et al,^11^ and the *z* ≥ 3 was used as the cutoff value for T21 detection. A set of 272 clinical euploid samples, which had been tested negative by both *z*‐score and DV approach, were used as the reference database (db). Window‐wise coverage fluctuation was estimated using a fixed 50‐kb bin along the non‐repetitive segments of the genome and window‐based *z*‐scores were calculated and their standard deviation (ZsdNorm) were estimated individually for each library to assess the evenness and randomness of the chromosome coverage. Additionally, the non‐reference‐based derivative value (DV) approach was used as described in Qi et al.^11^ In brief, the DV value was calculated as the derivative value of the estimated ploidy number divided by the estimated chromosomal copy number and the cutoff value of 1.05 was used to detect trisomy 21.

### 2.3 Statistical analysis

The differences and associations between groups were examined using ANOVA test/chi‐square test and Pearson's correlation coefficient respectively by SPSS software (version 22, IBM). A significant *P*‐value was defined as .05.

## 3 RESULTS

### 3.1 The effects of low total cfDNA content in NIPT analysis

**Figure 1:**
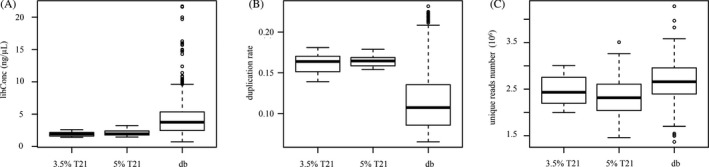
Comparison of sequencing attributes between experimental groups and reference database. A, the library concentration of samples in both 3.5% T21 and 5% T21 groups was significantly less than that in db; B, the duplication rate in both 3.5% T21 and 5% T21 groups was significantly higher than that of db group; C, a slight decrease in unique reads number in 3.5% T21 and 5% T21 groups; db represented database

To simulate the low total cfDNA scenario, 70% (v/v) PBS buffer was added to mock T21 samples and the concentration of each sample was on average only 30% of the normal concentration. After library construction, the average library concentration (LibConc) of 3.5% T21 group was 1.914 ng/μL ± 0.317, the LibConc of 5% T21 group was 2.097 ng/μL ± 0.411, and the reference database was 4.636 ng/μL ± 3.56 (Figure 1A). Among the total of 36 mock T21 samples, one sample in 3.5% T21 group failed to generate any usable reads, but the rest of samples in the same group (17/18) and all samples in 5% T21 group (18/18) were successfully sequenced.

In terms of the sequencing quality metrics, the results showed that comparing with the reference database, duplication rate was significantly increased in both 3.5% T21 and 5% T21 groups (*P* = 2.5836E‐13 and 2.36E‐30, respectively) and unique reads number was significantly decreased (*P* = .013 and 0.028, respectively) (Figure 1B,C). However, the differences between 3.5% T21 and 5% T21 groups were insignificant. This indicated that the deviation observed in sequencing data was caused by the low total cfDNA content only and it was independent of the fetal fraction.

### 3.2 The abnormal correlation between LibConc and sequencing attributes in samples with low total cfDNA

**Figure 2:**
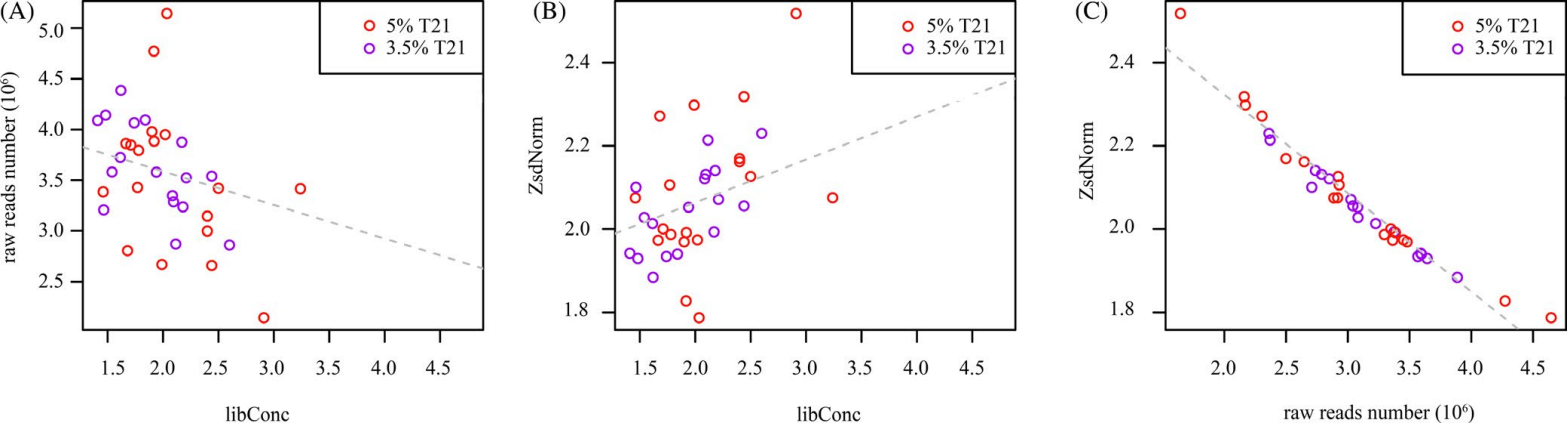
Relation of raw reads numbers, overall variation, and measured library concentration by Qubit testing system. A, the measured library concentration of mock samples and its raw reads number in 3.5% T21 and 5% T21 groups showed a negative correlation; B, the measured library concentration of mock samples and its window‐wise normalization score (ZsdNorm) in 3.5% T21 and 5% T21 groups presented a positive correlation; C, the raw reads number of mock samples and its ZsdNorm in 3.5% T21 and 5% T21 groups presented a strong negative correlation. Red circles present the samples in 5% T21 group, and purple circles presented the samples in 3.5% T21 group

In the study, an unexpected negative correlation was observed (Pearson's correlation coefficient = −0.4439637) between the measured library concentration of mock samples and their raw reads numbers, regardless of the fetal fraction (Figure 2A). This showed that higher LibConc resulted in a decreased quantity of raw sequencing reads, which was opposite to the common understanding. In addition, a positive correlation (Pearson's correlation coefficient = 0.5036082) was found in both 3.5% T21 and 5% T21 groups (Figure 2B) between the LibConc and the variation of window‐wise normalization score ZsdNorm, an indicator of the randomness and evenness in the overall coverage. This exhibited that higher LibConc generated higher inter‐chromosomal variation of coverage, which was also a surprising observation. Lastly, a negative correlation (Pearson's correlation coefficient = −0.9749898) was obtained between raw reads count and ZsdNorm (Figure 2C), demonstrating that more reads would lead to a better coverage through higher sequencing depth, which was consistent with the common knowledge.

### 3.3 The Derivative value approach could increase the T21 detection rate in samples with low total cfDNA

**Figure 3:**
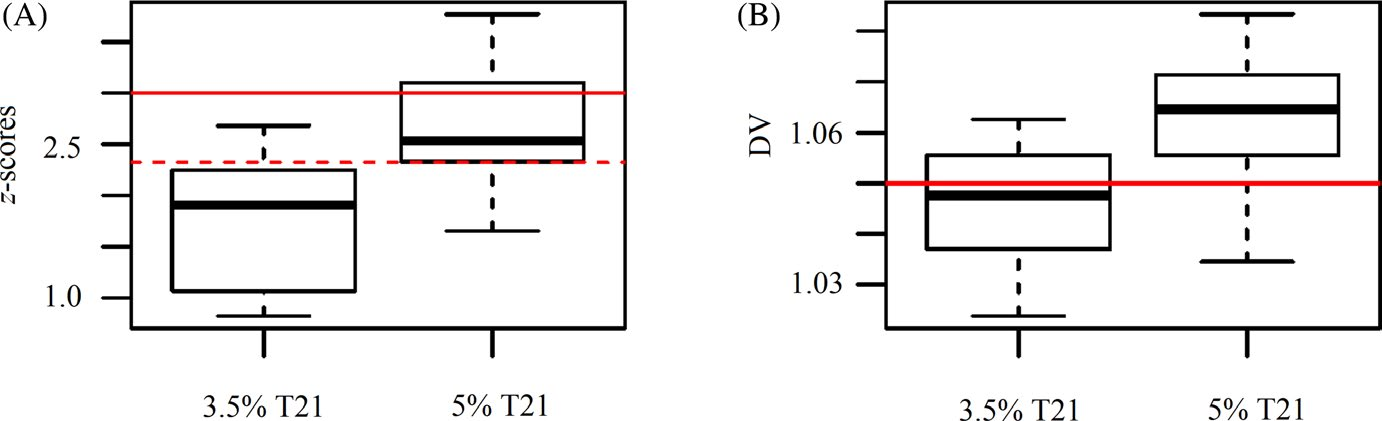
Different FF ratio in low total cfDNA content affected the NIPT results. A, results of the *z*‐score method in 3.5% T21 and 5% T21 groups, the horizontal red line indicated the cutoff value was 3 and the horizontal red dash line represented the cutoff value was 2.326384; B, results of the DV method in 3.5% T21 and 5% T21 groups, the horizontal red line indicated the cutoff value was 1.05.

In terms of the T21 detection, the data showed that all 17 samples from the 3.5% T21 group and 12 of 18 samples in the 5% T21 group had the *z*‐score below the cutoff value. However, after dropping the cutoff line to 2.33, which is the value of the 99% percentile in the reference db, the 3.5% FF and 5% FF group achieved a positive detection rate of 23.52% (4/17) and 77.78% (14/18), respectively (Figure 3A). As for the DV method, a theoretical cutoff value of 1.05 was used, 17 of 18 samples (94.44%) were successfully identified as T21 positive in the 5% T21 group, and 6 of 17 samples (35.29%) from the 3.5% T21 group were correctly confirmed (Figure 3B).

The performance of the *z*‐score method and the DV approach was compared using the chi‐square test. In samples with 5% fetal fraction, the DV approach was significantly different from the *z*‐score method (*P* = .00052), whereas the difference in 3.5% FF group was not so obvious between the two approaches (*P* = .0245). Thus, our results demonstrated that, at least in our test runs, the proposed DV approach achieved comparable and in certain cases better prediction, when fetal fraction was sufficiently high. However, in the cases of samples with low fetal fraction, low library DNA concentration might still be a threatening factor regardless of the analytical methods.

## 4 DISCUSSION

NIPT has been widely accepted in clinical practice for chromosome aneuploidy screening in the past years, and it has achieved much higher accuracy than the conventional serological prenatal screening for Down's syndrome and Edwards syndrome.^12^, ^13^, ^14^ However, a variety of factors were recently found to have significant impacts on the outcome of the test, among which, fetal fraction was considered to be most critical for the accuracy of NIPT.^15^, ^16^, ^17^ It is generally considered as unsuitable for NIPT screening in most commercial services when FF is lower than 4%.^18^ In this study, we used a more stringent value of 3.5% as the threshold for LFF, which was demonstrated meaningful in the previous study.^19^ In addition to LFF, sample's total cfDNA content was also considered as an important factor, but its effect on NIPT analysis and effects on low FF and normal FF samples have not been investigated. Thus, unlike others, we attempted to improve the results of NIPT in low total cfDNA samples and the findings were interesting.

Our data showed that the reduction in sample cfDNA content could be reflected by the decreased library concentration, increased duplication rate, and decreased unique reads count. However, its impact on LFF and 5% FF samples seemed to be equal, the differences between the two groups were not significant (Figure 1B,C). In addition, the negative relationship between LibConc and raw reads number and positive relationship between LibConc and ZsdNorm found in samples with low total cfDNA content were totally unexpected (Figure 2A,B). In theory, higher library concentration should be associated with higher raw reads count and less inter‐chromosomal variation of coverage, but in our cases, libraries with higher LibConc were accompanied with lower raw reads number and increased randomness in chromosome coverage. One possible explanation was that the Qubit 3.0 platform we used during the sample preparation overestimated the library concentration since it quantified DNA with fluorophores, which was not a direct measurement. The binding affinity of fluorophores to nucleic acid in the sample might vary, due to topological structure of the DNA, and also unwanted substances like proteins and lipids might affect the fluorescence signal. Therefore, the actual DNA content of overestimated samples was limited which resulted in the unexpected relationship between LibConc and raw reads number or ZsdNorm.

To increase the detection rate of the low concentration mock T21 samples, we carried out two approaches. The first approach was to lower the cutoff value from *z* ≥ 3 to 2.33. The effect of this adjustment was different for 3.5% T21 and 5% T21 samples. In 5% T21 group, the detection rate was increased from 33.33% to 77.78%, but in 3.5% T21 group, the change was not obvious. The DV method, on the other hand, worked well for 5% T21 group and significantly increased the detection rate to 94.44% (17/18), but unfortunately for the 3.5% T21 group, although the detection rate was uplifted from 0% to 35.29% (6/17), the predicting power was still overall unsatisfactory to suit the clinical needs. This result demonstrated that the DV method could be used as an alternative approach for T21 screening to reduce the incidence of “no‐call” when average sample library concentration was as low as 2 ng/μL and the fetal fraction was no less than 5%. However, the lowest boundary of the total cfDNA content for DV method to work was not tested in this study and should be investigated through a set of serial diluted samples. Clinical evaluation with a large population set should also be carried out before adapting it into the routine clinical practice in the future.

In summary, we investigated the impact of low total cfDNA content on the NIPT sequencing process and assessed its effects on LFF and normal FF samples. The quantity of cfDNA was found to be a key factor in T21 detection since decreased total cfDNA would cause the increased level of duplication and decreased detection rate when using the *z*‐score approach. However, the non‐reference‐based DV method could potentially compensate this problem on samples with FF no less than 5%. In addition, we discovered that the Qubit platform could sometime overestimate the library concentration in samples with low total cfDNA and this often led to a reduced total reads number and increased fluctuation in coverage. Therefore, the relationship between LibConc and raw reads number or ZsdNorm should be assessed as a means of precaution against overestimated samples before initiating the NIPT bioinformatics analysis.

## CONFLICT OF INTEREST

The authors declare that they have no competing interest.
